# Kashin–Beck Disease: A Risk Factor for Sarcopenia and Its Interaction with Selenium

**DOI:** 10.3390/nu16244343

**Published:** 2024-12-16

**Authors:** Haotian Wu, Zhaoyu Chen, Ou Wang, Tong Jiang, Jian Huang, Jun Wang, Jianhao Lin

**Affiliations:** 1 Peking University Arthritis Clinic and Research Center, Peking University People’s Hospital, Xicheng, Beijing 100044, China; wu_ht@pku.edu.cn (H.W.); czy41@live.com (Z.C.); 2 Key Laboratory of Trace Element Nutrition of National Health Commission of People’s Republic of China, National Institute for Nutrition and Health, Chinese Center for Disease Control and Prevention, Xicheng, Beijing 100050, China; wangou@ninh.chinacdc.cn (O.W.); jiangtong@ninh.chinacdc.cn (T.J.); huangjian@ninh.chinacdc.cn (J.H.); 3 School of Food and Drug, Shenzhen Polytechnic University, No. 7098 Liuxian Avenue, Nanshan, Shenzhen 518055, China; junwangwh@hotmail.com

**Keywords:** endemic musculoskeletal disease, nutrition-related disease, element concentration analysis, partial correlation analysis, multivariate regression analysis

## Abstract

Objectives: We aimed to explore the possible effects of Kashin–Beck disease (KBD) on the risk of sarcopenia and its possible interaction in the association between the risk of sarcopenia and element concentration. Methods: This cross-sectional study was conducted among individuals 18–75 years old in Qamdo, a KBD-endemic area. All individuals received physical and radiological examinations before recruitment. Patients with KBD were enrolled in the KBD group based on a diagnosis of national criteria WS/T 207-2010. Healthy individuals without KBD were enrolled in the non-KBD group. Participants with a history of element supplements, other severe musculoskeletal diseases, or organ dysfunctions were excluded. We adopted WOMAC scores for the assessment of musculoskeletal conditions and SARC-F scores for the risk of sarcopenia. Patients with SARC-F ≥ 4 were at risk of sarcopenia. Serum element concentrations were analyzed by inductively coupled plasma mass spectrometry. Dose–relationship effects of clinical scores and element concentrations on the risk of sarcopenia were determined in correlation analysis. Risk factors were identified using univariate and multivariate regression. Statistical analysis was conducted using R software. Results: A total of 65 patients with KBD and 38 participants without KBD were enrolled in the analysis. After propensity score matching, population characteristics were comparable in the two groups, and the incidence of SARC-F ≥ 4 was determined to be higher in the KBD group (*p* = 0.002). The WOMAC scores were correlated with SARC-F scores in the KBD group (*p* < 0.001) and non-KBD (*p* < 0.001) group, respectively. Further analysis proved that KBD was the independent risk factor for the risk of sarcopenia (*p* = 0.014). Moreover, high Selenium concentrations were associated with a low risk of sarcopenia in the non-KBD group (*p* = 0.047), while this association was not observed in the KBD group (*p* = 0.239). Conclusions: KBD as an independent risk factor increased the risk of sarcopenia for patients. Although high Se concentration was associated with a low risk of sarcopenia in participants without KBD, this association was not observed in those with KBD.

## 1. Introduction

Kashin–Beck disease (KBD) is an endemic chronic joint disease affecting approximately 0.09% of residents in Tibet, China [1,2]. This disease mainly affects the epiphyseal plate and articular cartilage of children. Although its etiology remains unclear, the incidence of KBD is found to be associated with several nutritional risk factors, including trace element deficiency, water organic compounds poisoning, and food fungi poisoning [3,4,5]. Patients suffer from extremely poor physical functions and joint deformities after long-term progression. In addition, malnutrition and deformities among patients with KBD increase the risk of other musculoskeletal disorders. Thus, an early investigation and intervention for musculoskeletal disorders within patients with KBD makes sense.

Sarcopenia is a common musculoskeletal complication with a higher incidence in the Qinghai–Tibet plateau population compared with the plain population [6,7]. Patients with sarcopenia complain about decreased muscle strength and restricted physical performance caused by muscular atrophy and fatty infiltration [8]. Recently, the revised European consensus published by the European Working Group on Sarcopenia in Older People (EWGSOP2) provided the approach for the diagnosis of sarcopenia, including a screening scale known as the Strength, Assistance with walking, Rise from a chair, Climb stairs, and Falls sarcopenia (SARC-F) score, a test of muscle strength, and a further evaluation of the quantity and quality of muscles [9]. For the initial screening of the risk of sarcopenia, the SARC-F score is widely adopted in published studies [10,11].

Until now, there have been no published studies on the relationship between KBD and the risk of sarcopenia. Recently, patients with osteoarthritis have proved to be at higher risk of sarcopenia [6]. In addition to the nutritional factors, functional loss and chronic pain in patients with osteoarthritis are considered to be related to the progression of sarcopenia [12]. Sarcopenia has a significant impact on clinical outcomes in patients with musculoskeletal disorders, with an elevation of falls and other terrible events [13]. Considering that the characteristics are frequently seen in patients with KBD as well, a further investigation of the risk of sarcopenia is beneficial for the management of KBD [7].

Moreover, the progression of both KBD and sarcopenia are considered to be associated with element concentrations. For KBD, the deficiency of Selenium (Se) is frequently observed in endemic areas, and this low Se concentration has been proposed as an important risk factor in KBD incidence [14]. For sarcopenia, according to data from the National Health and Nutrition Examination Survey, higher dietary Se intake (≥80.10 μg/day) is associated with a lower incidence of sarcopenia in adults [15]. In addition, Calcium (Ca) and Zinc (Zn) deficiencies and elevated Copper (Cu) content are also proven to be related to the pathogenesis of sarcopenia [16,17,18]. However, previously published studies uncovering the associations between elements and sarcopenia have focused on individuals without KBD. Whether the associations exist in patients with KBD remains uncertain.

In view of this, we delivered this cross-sectional study in Tibet, China, aiming to (1) evaluate the risk of sarcopenia for patients with KBD, (2) explore the risk factors for sarcopenia, and (3) explore the possible interaction of KBD in the association between the risk of sarcopenia and element concentration.

## 2. Materials and Methods

### 2.1. Participant Eligibility

A cross-sectional study enrolling patients with KBD and participants without KBD was conducted by the National Institute for Nutrition and Health (NINH) of the Chinese Center for Disease Control and Prevention (CCDC) and Peking University People’s Hospital (PKUPH) in Qamdo, Tibet, China, in November 2021. The participant recruitment was conducted in KBD historical endemic areas according to national criteria (GB 16395-2011) [19]. For the KBD group, we enrolled the eligible patients after physical and X-ray examinations, following the inclusion criteria: (1) patients diagnosed as KBD according to national diagnostic criteria WS/T 207-2010 [20], (2) patients 18–75 years old, and (3) permanent residents who lived in Qamdo for the past six months. As for the non-KBD group, we randomly enrolled participants from healthy individuals after physical and X-ray examinations in the same KBD historical endemic areas, with the following criteria: (1) participants without KBD, (2) participants 18–75 years old, and (3) permanent residents who lived in Qamdo for the past 6 months. The participants would be excluded if they had a (1) history of elements supplement or medication for KBD during the past six months; (2) reports of other musculoskeletal disorders, including severe degenerative osteoarthritis (Kellgren–Lawrence grade 3–4), rheumatoid arthritis, traumatic arthritis, and others resulting in severe deformities; (3) severe cardiovascular, hepatic, or renal dysfunction causing severe difficulties in daily activities; and (4) unwilling intention to participate in this study. The data and sample collection began right after the recruitment of participants, and the evaluation and statistical analysis started in December 2021. All participants were informed about the researchers, objective, study design, inclusion and exclusion criteria, individual data collection including physical and radiological examination, survey and serum sample acquisition, data protection, and potential benefit and hurt to participants, and participants with any concerns on this study could withdraw at any period of this study. This study was conducted in accordance with the Declaration of Helsinki and approved by the Institutional Review Board of the National Institute for Nutrition and Health of the Chinese Center for Disease Control and Prevention (No. 2021-009, approval date: 2 April 2021). Written informed consent was obtained from all the patients to publish this paper. The report of our study followed the Strengthening the Reporting of Observational Studies in Epidemiology (STROBE) statement.

### 2.2. KBD Diagnosis

The diagnosis of KBD was made by an orthopedic specialist from PKUPH, following the national criteria WS/T 207-2010 [21]. KBD patients presented functional loss; pain and stiffness with thickening of fingers, toes, and other joints; and short deformities in a physical examination. In our study, X-ray images of hands and ankles were taken. Multiple symmetrical radiological changes of bone sclerosis, depression, destruction, and deformation could be detected in metaphyseal pre-calcification zones [22]. When other related musculoskeletal disorders were excluded, patients presenting characteristic symptoms and radiological changes with a history of living in KBD-epidemic areas for >6 months could be diagnosed as KBD.

### 2.3. Data Collection

For participants in the KBD group and non-KBD group, the population characteristics were collected, including sex, age, (BMI), experience of school education, occupation, yearly income (per capita in family). The sex, age, experience of school education, occupation, yearly income, and physical activity were recorded via survey. The height and weight of participants were measured by two clinicians from PKUPH. The height was measured to the nearest 0.1 cm using a height gauge while participants were standing upright, and the weight was measured to the nearest 0.1 kg using an electronic scale without shoes and in light clothing [23]. The body mass index (BMI) was calculated by weight(kg)/height(m)^2^. The Western Ontario and McMaster Universities osteoarthritis index (WOMAC) was adopted for the assessment of musculoskeletal condition, which consisted of three domains including functions, pain, and stiffness [24,25]. The full score for the WOMAC was 96, and WOMAC > 40 was considered as a poor musculoskeletal condition [26,27]. As for the assessment of the risk of sarcopenia, the SARC-F score was evaluated [10]. Patients with SARC-F ≥ 4 were considered to be at risk of sarcopenia, and a higher SARC-F score indicated a higher risk of sarcopenia [28,29].

### 2.4. Elemental Analysis of Blood Samples

In our study, the blood samples were obtained from all the participants in the KBD group and non-KBD group, and the elemental analysis was conducted by NINH. After digestion with 65% HNO_3_ in a microwave, the cooled and digested samples were diluted and analyzed for the concentrations of Ca, Se, Zn, and Cu by inductively coupled plasma mass spectrometry (ICP-MS) [30]. We used the medians of element concentrations before propensity score matching (PSM) as the cut-off values for high (≥median) and low (<median) serum element levels (Table S1) [31,32,33].

### 2.5. Statistical Analysis

For population characteristics, the descriptive statistics between the KBD group and non-KBD group were analyzed. For categorical variables, a chi-square test was performed. For continuous variables, a *t*-test was performed for variables corresponding to a normal distribution (mean value ± standard deviation [SD]), and the Mann–Whitney U test was performed for variables corresponding to a non-normal distribution (median value with interquartile range [IQR]) [34]. The 1:1 PSM for age and sex (caliper = 0.20) was adopted to make the population characteristics comparable between the KBD group and non-KBD group [35]. The correlation and partial correlation analysis of WOMAC scores and element concentrations with SARC-F scores were conducted in model A (non-adjusted), model B (age- and sex-adjusted), and model C (fully adjusted by age, sex, BMI, experience of school education, occupation, and yearly income) [36,37]. The univariate and multivariate logistic regression analysis were used to identify the independent risk factors [38]. The continuous variables were converted to categorical variables for the applicability of analysis. A value of *p* < 0.05 was considered as a significant difference. R software (version 4.4.1) was utilized for statistical analysis in our study. The post hoc test for effect size was calculated by PASS (version 15). 

### 2.6. Potential Sources of Bias

In our study, the potential sources of bias came from several points, including the limited number of participants, the attrition, and the limited assessment method for risk of bias. Firstly, the number of participants enrolled in our study was limited, and there might be some bias between population characteristics among participants in this study and residents living in Qamdo, Tibet. Secondly, a potential source of bias might also come from attrition because of incomplete data collection in our study. Moreover, more evaluation for the quantity and quality of muscles in addition to the SARC-F score was not involved in this study. With more assessment methods involved in future studies, more global sights of sarcopenia with less bias might be detected in KBD patients and non-KBD participants.

## 3. Results

### 3.1. Population Characteristics

**Figure 1:**
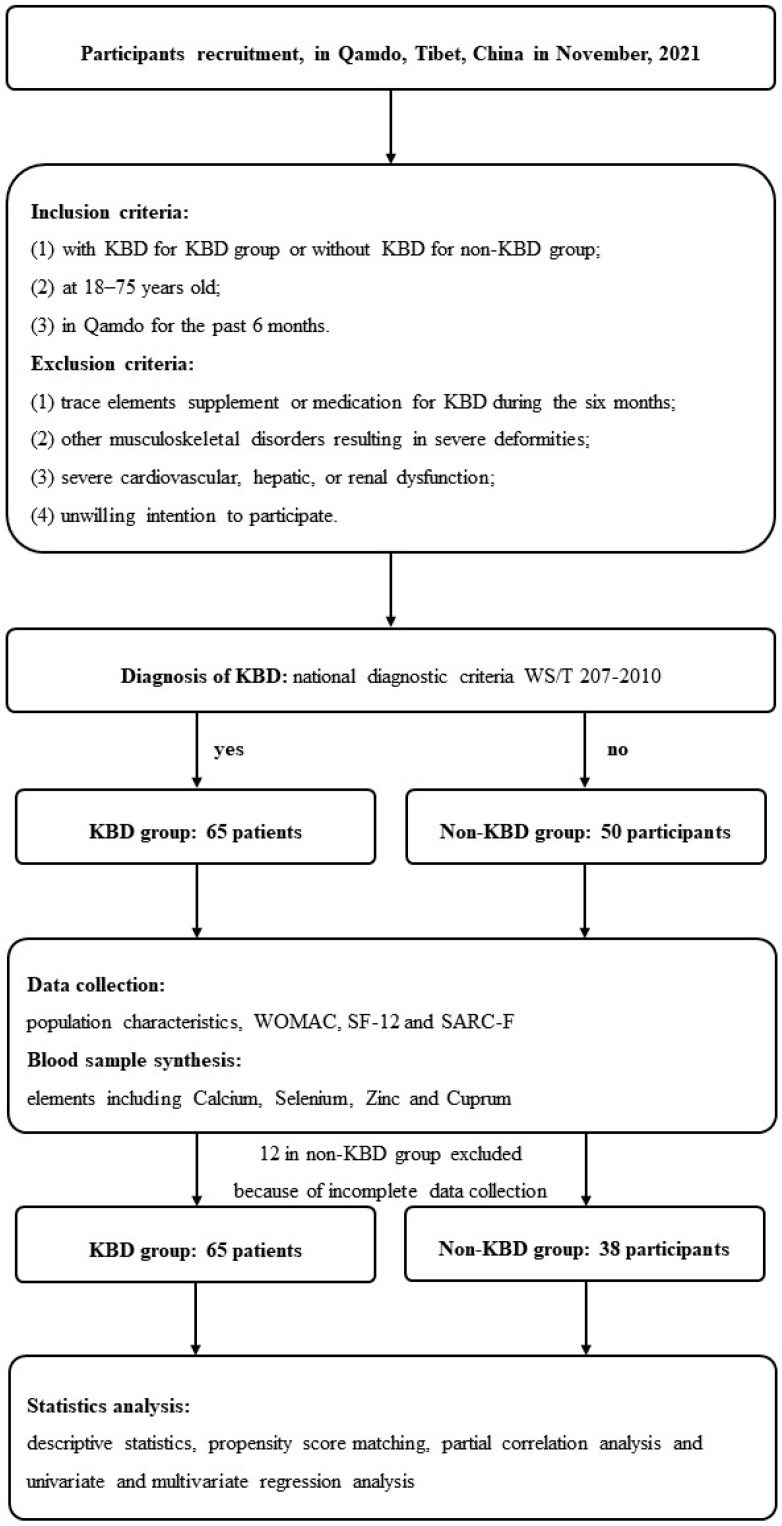
Flow chart of this study. KBD, Kashin–Beck disease; WOMAC, Western Ontario and McMaster Universities osteoarthritis index; SARC-F, Strength, Assistance with walking, Rise from a chair, Climb stairs and Falls sarcopenia score; Ca, Calcium; Se, Selenium; Zn, Zinc; Cu, Cuprum.

We enrolled 115 participants in our study, among whom 12 participants without KBD were excluded from the analysis because of incomplete data collection. Finally, a total of 103 participants were subjected to statistical analysis, including 65 patients in the KBD group and 38 participants in the non-KBD group (Figure 1). As shown in Table 1, there were significant differences between the two groups in age and sex (Table 1). After 1:1 PSM (caliper = 0.20) for age and sex, all of the baseline characteristics were comparable between the KBD group and non-KBD group (Table 2). Among the participants after PSM, the mean age was 43.8 ± 10.2 y, and the proportion of females was 31.0%. The median BMI was 23.4 (3.8) kg/m^2^. In addition, 60.3% of participants had not received school education; 79.3% of patients were farmers or herdsmen with physically demanding work and 62.1% of participants declared earning less than 10,000 Chinese yuan (CNY) per year.

### 3.2. Clinical Comparisons Between KBD and Non-KBD Participants

It was remarkable that patients with KBD exhibited extremely worse musculoskeletal condition compared with participants without KBD. Compared with participants without KBD, patients with KBD reported more difficulties in joint functions, pain, and stiffness through the self-assessment of WOMAC scores (Table 3). According to the WOMAC scores, 73.8% (48/65) of patients with KBD had a poor musculoskeletal condition (WOMAC > 40), while only 39.5% (15/38) of participants without KBD had a poor condition (*p* = 0.001). The subgroup analysis between age (≥50 vs. <50 years old), sex (male vs. female), or BMI (≥18.5 vs. <18.5) groups presented no significant differences in the KBD group or in the non-KBD group (Table S2). When it comes to the risk of sarcopenia, the proportion of participants with the risk of sarcopenia was higher in the KBD group compared with the non-KBD group. The trends for clinical scores were consistent after PSM (Table 3).

The medians and IQRs for element concentrations are listed in Table S1. Patients with KBD had lower serum concentrations of Ca, Se, and Zn and a higher concentration of Cu. There were no significant differences between groups after PSM for age and sex.

### 3.3. Dose–Response Effect on SARC-F Scores

For all participants enrolled in this study, correlation analysis and partial correlation analysis were adopted for possible dose–response effects on SARC-F scores. In our analysis, higher WOMAC scores for function, pain, and stiffness were correlated with higher SARC-F scores in the KBD group and non-KBD group (Table 4). The significant correlations were preserved in both KBD patients and non-KBD participants when the model was adjusted by age and sex in model 2 and by age, sex, BMI, experience of school education, occupation, and yearly income in model 3. 

**Figure 2:**
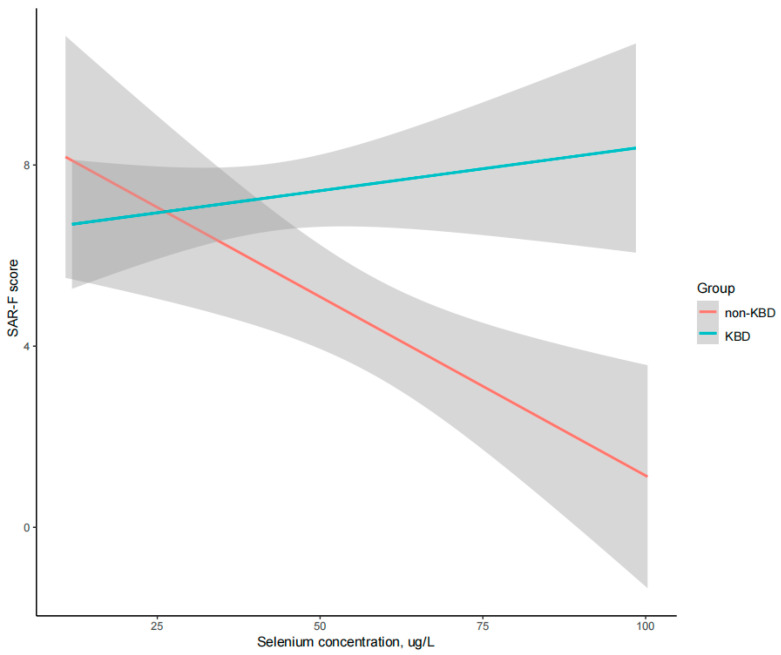
Correlation between Se concentrations and SARC-F scores. KBD, Kashin–Beck disease; SARC-F, Strength, Assistance with walking, Rise from a chair, Climb stairs and Falls sarcopenia score; Se, Selenium.

When it comes to the element concentration, a higher Se concentration was negatively correlated with a lower SARC-F score in participants without KBD (Table 5). However, the Se concentration was not correlated with the SARC-F score in patients with KBD (Figure 2). These findings indicate that there might be an interaction of KBD in the association between sarcopenia and Se concentration. Moreover, there was also a significant correlation between sarcopenia and Ca concentration, though the significance was not observed when all variables were adjusted in model C.

### 3.4. Univariate and Multivariate Regression Analysis for Risk of Sarcopenia

After the univariate regressions for all patients enrolled in this study, KBD and age ≥ 50 years were risk factors for the risk of sarcopenia. The factors with *p* < 0.10 in univariate regression were selected into the multivariate regression analysis, and KBD was the only independent risk factor for risk of sarcopenia with SARC-F ≥ 4 (odds ratio [OR] = 3.64; 95%CI 1.30, 10.21; *p* = 0.014) (Table 6). In our analysis, the effect size reached 0.84 for KBD as an independent risk factor for the risk of sarcopenia, calculated by PASS.

In our analysis, a high Se level was associated with a low risk of sarcopenia (OR = 0.39, 95%CI 0.16, 0.95, *p* = 0.038). Further univariate regression analysis in non-KBD participants presented that a high Se level was associated with a low risk of sarcopenia (OR = 0.70, 95%CI 0.50, 0.98, *p* = 0.047), while this association was not detected in KBD patients (OR = 0.84, 95%CI 0.63, 1.12, *p* = 0.239).

## 4. Discussion

Both KBD and sarcopenia are harmful musculoskeletal disorders threatening the quality of life of patients in Tibet [39,40]. However, there were no published comorbidity studies examining the association between KBD and sarcopenia. Our work was the first study to identify the role of KBD in sarcopenia, reminding clinicians to estimate the risk of sarcopenia during KBD management.

In our study, KBD was an independent risk factor for the risk of sarcopenia. Possible explanations were the functional impairment and pain in KBD patients. In our study, patients with KBD presented heavier functional impairment and worse pain in the musculoskeletal system. Patients with functional problems in lower limbs were proven to be at higher risk of muscle atrophy and fatty infiltration [12,41]. For example, a study on hip osteoarthritis indicated the relationship between WOMAC scores for function and stiffness and muscle atrophy and fatty infiltration, which were frequently detected in sarcopenia [12,42]. Moreover, pain, especially chronic persistent pain, would increase the risk of sarcopenia in middle-aged and old adults [43]. A possible explanation was that long-term pain kept the muscles in an inflammatory environment with various elevated inflammatory factors. An increased level of interleukin (IL)-6 induces the loss of muscle strength and mass, possibly due to the IL-6/sIL-6R pathway [44]. Tumor necrosis factor (TNF)-α also has a negative effect on muscles by promoting the degradation and downregulating the synthesis of proteins, as well as promoting the release of IL-6 [44,45]. In addition, chronic pain frequently leads to a decrease in tolerance of activities, and inflammation in arthritis causes the degeneration of neuromuscular junctions, representing further risk factors for sarcopenia [46,47]. However, mechanism studies on the possible effects of functional impairment and pain in sarcopenia had not been conducted among patients with KBD yet. More direct evidence on mechanisms was expected to uncover the relationship between the progression of KBD and sarcopenia.

Our results in the non-KBD group indicated an association between the risk of sarcopenia and Se concentration. Previously published studies suggested that participants with a higher Se concentration were in a better nutritional condition and were at a lower risk of sarcopenia [48]. A possible explanation was selenoproteins. These Se-dependent antioxidant proteins are important for allowing muscles to escape from oxidative damage by oxygen and nitrogen species, which is considered to be important in the progression of sarcopenia [16,49]. Among a variety of selenoproteins, glutathione peroxidases play a key role in muscular antioxidant effects [50]. Besides glutathione peroxidases, other selenoproteins regulate the biogenesis and functions of mitochondria in muscle cells, resulting in the mass and strength reduction of muscles. For example, selenoprotein O enhances mitochondrial biogenesis, and selenoproteins N and W influence the mitochondrial function in muscles by the regulation of Ca homeostasis [15]. Moreover, we also noticed the relationship between Ca concentration and the risk of sarcopenia. A possible explanation for the effect of Ca on sarcopenia was the modulating effect of calpains [51]. The Ca-dependent cysteine proteases play a significant role in the regulation of cell adhesion, migration, and fusion during myogenesis [51]. Although there have been studies on the possible mechanisms of elements in the regulation of muscle health, more evidence in epidemiology and in the laboratory was required to further identify the effect of elements in the progression of sarcopenia.

Meanwhile, associations between SARC-F scores and Se concentrations were not detected among KBD patients in our study. KBD presented an interaction in the possible effect of Se on the risk of sarcopenia. Our further correlation analysis found that higher Se was correlated with lower WOMAC scores in patients without KBD, while this association was not detected in the KBD group (Table S3). A possible explanation was that functional impairment and chronic pain played an important role in the risk of sarcopenia in patients with KBD, and a high Se concentration failed to improve the functions and pain for adult patients with years of disease history after epiphyseal closure. Previous studies presented that functional loss and chronic pain in osteoarthritis were strongly associated with sarcopenia [12,46,52]. These findings supported our hypothesis, and further prospective investigations into the association between sarcopenia and KBD are expected. Moreover, Se supplementation was proven to improve musculoskeletal conditions by providing protection for the growth of epiphyseal plates against oxidative damage among children in KBD-endemic areas [14]. However, there was no evidence supporting that Se supplementation could improve functions and pain in adult patients who had a severe deformity with already closed epiphyseal plates. Although further studies in the laboratory are required for the interaction of KBD in the association between sarcopenia and Se in adult patients, our study reminds clinicians to pay attention to the risk of sarcopenia in the management of KBD even if patients have a relatively high level of Se.

Although this study initially identified KBD as an independent risk factor for the risk of sarcopenia, several limitations still existed in our work. Firstly, considering that this was a cross-sectional study, the long-term progression in the risk of sarcopenia was unavailable. Prospective cohort studies are required for the further assessment of the effect of KBD on other musculoskeletal disorders. Secondly, the number of eligible participants was limited. With the rapid development of road conditions, medical equipment, and strategies for the management of KBD, more participants are expected in future studies on patients with KBD in Tibet. In addition, further evaluations for the quantity and quality of muscles are expected in future analysis, as the EWGSOP2 suggests, for a more accurate assessment of sarcopenia. Moreover, the hair Se concentration was not detected in this study. Although the Se concentrations in both serum and hair reflect the long-term Se level in the body, the serum Se concentration is believed to be more susceptible to dietary changes or infectious diseases [53]. The hair Se concentration can be sampled non-invasively and is more stable compared with the serum Se concentration, which could help in obtaining more convincing results.

## 5. Conclusions

Our study initially identified KBD as an independent risk factor for the risk of sarcopenia. Among patients with KBD, the WOMAC scores for function, pain, and stiffness were correlated with the SARC-F scores. Moreover, a high Se concentration was associated with a low risk of sarcopenia among individuals without KBD in KBD-endemic areas. An interaction of KBD in the association between risk of sarcopenia and Se concentration was not detected in patients with KBD. Our findings call for more attention to the risk of sarcopenia in the management of KBD.

## Figures and Tables

**Table 1:** Participant characteristics.

	Total	KBD Group	Non-KBD Group	*p* Value
Participant number	103	65	38	-
Mean age ± SD, years	46.4 ± 12.2	50.8 ± 10.8	38.9 ± 10.8	<0.001
Female, n (%)	41 (39.8)	32 (49.2)	9 (23.7)	0.011
Median BMI (IQR), kg/m^2^	22.5 (4.3)	22.5 (4.9)	22.6 (3.7)	0.806
School education, n (%)	38 (36.9)	21 (32.3)	17 (44.7)	0.207
Occupation, n (%)				0.536
Farmers and herdsmen	87 (84.5)	56 (86.2)	31 (81.6)	
Others	16 (15.5)	9 (13.8)	7 (18.4)	
Yearly income, n (%)				0.115
<10,000 CNY	63 (61.2)	36 (55.4)	27 (71.1)	
≥10,000 CNY	40 (38.8)	29 (44.6)	11 (28.9)	

KBD, Kashin–Beck disease; SD, standard deviation; IQR, interquartile range; BMI, body mass index; CNY, Chinese yuan.

**Table 2:** Participant characteristics after 1:1 PSM.

	Total	KBD Group	Non-KBD Group	*p* Value
Participant number	58	29	29	-
Mean age ± SD, years	43.8 ± 10.2	45.8 ± 10.7	41.9 ± 9.5	0.148
Female, n (%)	18 (31.0)	10 (34.5)	8 (27.6)	0.570
Median BMI (IQR), kg/m^2^	23.4 (3.8)	23.7 (5.4)	23.4 (3.2)	0.253
School education, n (%)	23 (39.7)	10 (34.5)	13 (44.8)	0.421
Occupation, n (%)				0.517
Farmers and herdsmen	46 (79.3)	24 (82.8)	22 (75.9)	
Others	12 (20.7)	5 (17.2)	7 (24.1)	
Yearly income, n (%)				0.279
<10,000 CNY	36 (62.1)	16 (55.2)	20 (69.0)	
≥10,000 CNY	22 (37.9)	13 (44.8)	9 (31.0)	

KBD, Kashin–Beck disease; PSM, propensity score matching; SD, standard deviation; IQR, interquartile range; BMI, body mass index; CNY, Chinese yuan.

**Table 3:** Comparison of health scores between KBD and non-KBD group before and after PSM.

	Before PSM			After PSM		
	KBD	Non-KBD	*p* Value	KBD	Non-KBD	*p* Value
Median WOMAC (IQR)						
WOMAC total	59 (56)	10 (58)	<0.001	68 (54)	27 (61)	0.001
WOMAC function	41 (44)	6 (39)	<0.001	46 (40)	16 (40)	0.001
WOMAC pain	16 (11)	3 (12)	<0.001	16 (10)	6 (13)	<0.001
WOMAC stiffness	6 (5)	1 (6)	<0.001	6 (6)	1 (7)	0.009
Median SARC-F (IQR)	8 (6)	3 (7)	<0.001	9 (5)	4 (8)	0.001
Risk of sarcopenia, n (%)	55 (84.6)	18 (47.4)	<0.001	26 (89.7)	15 (51.7)	0.002

KBD, Kashin–Beck disease; PSM, propensity score matching; IQR, interquartile range; WOMAC, Western Ontario and McMaster Universities osteoarthritis index; SARC-F, Strength, Assistance with walking, Rise from a chair, Climb stairs and Falls sarcopenia score.

**Table 4:** Correlation analysis and partial correlation analysis of WOMAC scores with SARC-F scores.

	KBD Group			Non-KBD Group		
WOMAC	Function	Pain	Stiffness	Function	Pain	Stiffness
Model A						
correlation coefficient	0.874	0.706	0.652	0.949	0.954	0.967
*p* value	<0.001	<0.001	<0.001	<0.001	<0.001	<0.001
Model B						
correlation coefficient	0.869	0.692	0.652	0.948	0.956	0.968
*p* value	<0.001	<0.001	<0.001	<0.001	<0.001	<0.001
Model C						
correlation coefficient	0.865	0.657	0.607	0.952	0.955	0.969
*p* value	<0.001	<0.001	<0.001	<0.001	<0.001	<0.001

Model A, non-adjusted correlation analysis; model B, age- and sex-adjusted partial correlation analysis; model C, fully adjusted partial correlation analysis; KBD, Kashin–Beck disease; WOMAC, Western Ontario and McMaster Universities osteoarthritis index; SARC-F, Strength, Assistance with walking, Rise from a chair, Climb stairs and Falls sarcopenia score.

**Table 5:** Correlation analysis and partial correlation analysis of element concentrations with SARC-F scores.

	KBD Group				Non-KBD Group			
Element	Ca	Se	Zn	Cu	Ca	Se	Zn	Cu
Model A								
correlation coefficient	0.061	0.124	0.138	0.178	−0.335	−0.456	−0.211	−0.042
*p* value	0.627	0.326	0.274	0.155	0.040	0.004	0.203	0.803
Model B								
correlation coefficient	0.024	0.247	0.128	0.152	−0.336	−0.424	−0.170	−0.031
*p* value	0.850	0.051	0.318	0.234	0.045	0.010	0.322	0.856
Model C								
correlation coefficient	0.087	0.229	0.151	0.142	−0.340	−0.469	−0.244	−0.257
*p* value	0.512	0.080	0.253	0.284	0.057	0.007	0.179	0.155

Model A, non-adjusted correlation analysis; model B, age- and sex-adjusted partial correlation analysis; model C, fully adjusted partial correlation analysis; Ca, Calcium; Se, Selenium; Zn, Zinc; Cu, Cuprum.

**Table 6:** The univariate and multivariate logistic regression for the risk of sarcopenia.

	Univariate Regression			Multivariate Regression		
	OR	95%CI	*p* Value	OR	95%CI	*p* Value
Age (≥50 vs. <50), years	5.14	1.77, 14.89	0.003	2.86	0.87, 9.42	0.083
Sex (male vs. female)	1.01	0.42, 2.41	0.979			
BMI (≥18.5 vs. <18.5), kg/m^2^	0.70	0.18, 2.75	0.609			
School education (yes vs. no)	0.83	0.35, 1.99	0.675			
Occupation (farmers and herdsmen vs. others)	1.57	0.52, 4.81	0.425			
Yearly income (≥10,000 vs. <10,000), CNY	1.14	0.47, 2.74	0.772			
KBD (yes vs. no)	6.11	2.42, 15.44	<0.001	3.64	1.30, 10.21	0.014
Ca level (high vs. low)	0.48	0.20, 1.14	0.098	0.67	0.24–1.83	0.433
Se level (high vs. low)	0.39	0.16, 0.95	0.038	0.80	0.28, 2.29	0.682
Zn level (high vs. low)	0.85	0.36, 1.99	0.711			
Cu level (high vs. low)	1.82	0.77, 4.31	0.175			

KBD, Kashin–Beck disease; OR, odds ratio; 95%CI, 95% confidence interval; BMI, body mass index; CNY, Chinese yuan; Ca, Calcium; Se, Selenium; Zn, Zinc; Cu, Cuprum.

## Data Availability

The original contributions presented in this study are included in the article/Supplementary Materials. Further inquiries can be directed to the corresponding author.

## Supplementary Materials

The following supporting information can be downloaded at https://www.mdpi.com/article/10.3390/nu16244343/s1, Table S1: Comparison of serum element concentrations between KBD and non-KBD group before and after PSM. Table S2: Comparison of health scores between subgroups in KBD and non-KBD group. Table S3: Correlation analysis and partial correlation analysis of WOMAC scores with Se concentrations.
