# Supplementary material for: Kashin–Beck Disease: A Risk Factor for Sarcopenia and Its Interaction with Selenium
Source: Nutrients. 2024 Dec 16;16(24):4343. doi: 10.3390/nu16244343 (PMC11678709; doi:10.3390/nu16244343)
Supplement: Supplementary file 1 [file nutrients-16-04343-s001.zip › nutrients-3342843-supplementary.pdf]

Table S1. Comparison of serum element concentrations between KBD and non-KBD group before and after PSM

	Before PSM			After PSM		
**Median (IQR)**	**KBD**	**Non-KBD**	***p* value**	**KBD**	**Non-KBD**	***p* value**
Ca, mg/L	82.5 (11.1)	86.6 (10.1)	0.025	84.9 (15.2)	84.8 (12.3)	0.957
Se, ug/L	39.8 (28.3)	61.2 (28.4)	<0.001	40.5 (33.2)	59.7 (36.7)	0.401
Zn, ug/dL	67.0 (17.1)	79.1 (15.4)	<0.001	70.0 (21.0)	79.1 (15.2)	0.211
Cu, ug/dL	107.5 (27.0)	95.3 (25.5)	0.009	104.9 (23.8)	95.8 (26.7)	0.061

KBD, Kashin-Beck disease; PSM, propensity score matching; IQR, interquartile range; Ca, Calcium; Se, Selenium; Zn, Zinc; Cu, Cuprum.

Table S2. Comparison of health scores between subgroups in KBD and non-KBD group

	**Age (≥ 50 vs < 50 years)**	**Sex (male vs female)**	**BMI (≥ 18.5 vs < 18.5 kg/m^2)**
KBD group	p =0.052	p =0.410	p =0.716
Non-KBD group	p =0.146	p =0.761	p =0.571

KBD, Kashin-Beck disease; BMI, body mass index.

Table S3. Correlation analysis and partial correlation analysis of WOMAC scores with Se concentrations

	**KBD group**			**Non-KBD group**		
**WOMAC**	**function**	**pain**	**stiffness**	**function**	**pain**	**stiffness**
Model A						
correlation coefficient	0.074	-0.098	-0.121	-0.452	-0.445	-0.465
p value	0.559	0.437	0.338	0.004	0.005	0.003
Model B						
correlation coefficient	0.203	0.066	0.037	-0.408	-0.414	-0.420
p value	0.111	0.608	0.773	0.014	0.012	0.011
Model C						
correlation coefficient	0.168	0.020	-0.020	-0.428	-0.432	-0.449
p value	0.204	0.883	0.882	0.015	0.014	0.010

KBD, Kashin-Beck disease; WOMAC, Western Ontario and McMaster Universities osteoarthritis index.
